# Quantification of Urinary NGAL Using Volumetric Dried Urine Sampling

**DOI:** 10.3390/biomedicines14081790

**Published:** 2026-08-09

**Authors:** Susanne Sütterlin, Lena Carlsson, Ziad Amer, Sandra Näslund, Jesper von Groote, Anders Olof Larsson, Eva Tano

**Affiliations:** 1 Department of Women’s and Children’s Health, Pediatric Nephrology and Infectious Diseases (PNID) Research Group, Uppsala University, 75185 Uppsala, Sweden; ziad.amer@akademiska.se; 2 Department of Pediatric Emergency, Uppsala University Children’s Hospital, 75185 Uppsala, Sweden; 3 Department of Specialized Pediatrics, Pediatric Nephrology, Uppsala University Children’s Hospital, 75185 Uppsala, Sweden; 4 Department of Medical Sciences, Section of Clinical Chemistry, Uppsala University, 75185 Uppsala, Sweden; lena.carlsson@medsci.uu.se (L.C.); anders.larsson@akademiska.se (A.O.L.); 5 Department of Medical Sciences, Section of Clinical Microbiology, Uppsala University, 75185 Uppsala, Sweden; sandra.naslund@akademiska.se (S.N.); jespervongroote@gmail.com (J.v.G.); eva.tano@akademiska.se (E.T.)

**Keywords:** dried spot urine, neutrophil gelatinase-associated lipocalin (NGAL), capitainer qDBS, volumetric dried sampling, biomarker measurement, decentralized sampling

## Abstract

**Objective:** To evaluate the measurement of urinary neutrophil gelatinase-associated lipocalin (NGAL) from dried urine samples collected using Capitainer qDBS discs compared with liquid urine. **Methods:** Eighty urine samples from children aged 0–18 years were analysed. NGAL concentrations measured in liquid urine were compared with concentrations obtained from dried urine collected on Capitainer qDBS cards and eluted for analysis using a commercial ELISA. Method agreement was assessed using linear regression and Bland–Altman analysis. **Results:** Sixty-nine samples yielded measurable NGAL concentrations in both methods. Median NGAL concentrations were 8.72 ng/mL (IQR 3.97–20.88) in liquid urine and 8.48 ng/mL (IQR 3.28–20.11) in qDBS eluates. NGAL concentrations measured from qDBS correlated with liquid urine measurements with a strong linear relationship (R^2^ = 0.98). Method agreement was good. Median recovery of 95.4% indicated slightly lower concentrations in qDBS eluates. **Conclusions:** NGAL measurements obtained from volumetric dried urine sampling using Capitainer qDBS discs showed good agreement with liquid urine measurements.

## 1. Introduction

Neutrophil gelatinase-associated lipocalin (NGAL) is a biomarker of renal tubular injury and inflammation that can be measured in urine and has potential applications in pediatric nephrology. Urinary NGAL has been proposed as an early marker of acute kidney injury and of urinary tract inflammation [1,2]. Robust quantification of urinary NGAL is essential for its application in both clinical and research settings. While measurement in liquid urine is well established, it relies on conventional sample handling and transport systems, which may limit its use in settings requiring decentralized sampling, repeated measurements, or collection outside clinical environments. Reliable quantification depends not only on analytical performance but also on pre-analytical factors, including sample collection, handling, and transport [3].

Dried sample collection has emerged as an alternative approach. In dried spot sampling, small volumes of biological fluid are applied to absorbent media and dried, enabling transport and storage at ambient conditions. Widely used in blood-based analyses, this approach facilitates decentralized sampling while offering improved analyte stability, simplified transport, and reduced biohazard risk [4]. Dried urine spot sampling has been explored in selected applications, including toxicology and metabolite analysis, but remains less established than dried blood spot techniques. Several studies have demonstrated good agreement between dried and liquid urine for small molecules; however, data on the use of dried urine sampling for quantitative measurement of protein biomarkers remain scarce [5,6].

A key limitation of conventional dried spot techniques is variability in applied sample volume and spot homogeneity, which may affect quantitative accuracy, particularly in quantitative applications. Volumetric dried sampling devices, such as Capitainer qDBS cards (Capitainer AB, Solna, Sweden), have been developed to enable standardized collection of defined sample volumes and reduce such variability [7,8]. While volumetric dried sampling has been evaluated for blood-based biomarkers, its application to urine and urinary biomarkers remains largely unexplored.

The aim of this study was to evaluate the analytical agreement between NGAL measurements obtained from dried urine samples collected using Capitainer qDBS discs and measurements performed directly in liquid urine.

## 2. Materials and Methods

### 2.1. Sample Collection

Eighty urine samples were collected over a three-month period in 2024 from children aged 0–18 years seeking care at Uppsala University Hospital. The samples originated from routine clinical testing and represented leftover material not required for diagnostic purposes. Samples were analysed in anonymized form, and no selection was made based on kidney status. As only leftover clinical samples were used, informed consent was waived. The study was approved by the Regional Ethical Review Board in Uppsala (Dnr 01-367).

### 2.2. NGAL Analysis

NGAL measurements in liquid urine were compared with measurements from dried urine collected on Capitainer B50 qDBS sampling cards (Capitainer AB, Solna, Sweden). For the liquid reference method, 20 µL of urine was diluted with 180 µL phosphate-buffered saline containing Tween (PBS-Tween), corresponding to a 1:10 dilution; 200 µL of the diluted sample was added to the ELISA plate and stored at −20 °C until analysis.

For the qDBS method, 50 µL of urine was applied to each Capitainer qDBS 50 µL sample disc and allowed to dry overnight at room temperature. After drying overnight, each sampling disc was manually transferred using clean forceps to an individual microcentrifuge tube containing 500 µL PBS for elution. The discs were then incubated on a shaking platform for one hour to elute the dried samples. Subsequently, 200 µL of the eluate was transferred to an ELISA plate and stored at −20 °C until analysis.

NGAL concentrations were measured using the Human Lipocalin-2/NGAL DuoSet ELISA kit (R&D Systems, Minneapolis, MN, USA) according to the manufacturer’s instructions. Before analysis, 80 µL PBS containing 1% bovine serum albumin was added to the wells followed by 20 µL of thawed sample, resulting in a final dilution of 1:50. Absorbance was measured at 450 nm using a microplate reader, and concentrations were corrected for total dilution factor.

### 2.3. Statistical Analysis

NGAL concentrations measured in liquid urine were compared with concentrations obtained from qDBS eluates. Agreement analyses (linear regression and Bland–Altman analysis) were performed on samples with quantifiable NGAL concentrations in both methods. Linear regression was used to describe the relationship between concentrations. Passing–Bablok regression was performed as method-comparison analysis to assess constant and proportional bias between qDBS and liquid urine measurements. Bland–Altman analysis was performed on logarithmically transformed data to assess agreement across the concentration range. For recovery calculations, all samples were included, and concentrations below the quantification limit were treated as zero. The lower limit of quantification was defined based on the lowest point of the standard curve according to the manufacturer’s specifications (78 pg/mL). Statistical analyses and graphics were performed in R version 4.5.1 (R Foundation for Statistical Computing, Vienna, Austria).

## 3. Results

Eighty urine samples were analysed, of which sixty-nine yielded measurable NGAL concentrations with both sampling methods and were included in the agreement analyses. The median NGAL concentration measured directly in liquid urine was 8.72 ng/mL (IQR 3.97–20.88), compared with 8.48 ng/mL (IQR 3.28–20.11) for samples analysed from qDBS. Eleven samples had NGAL concentrations below the quantification limit in at least one method: 2 of 80 liquid urine samples and 9 of 80 qDBS samples.

**Figure 1:**
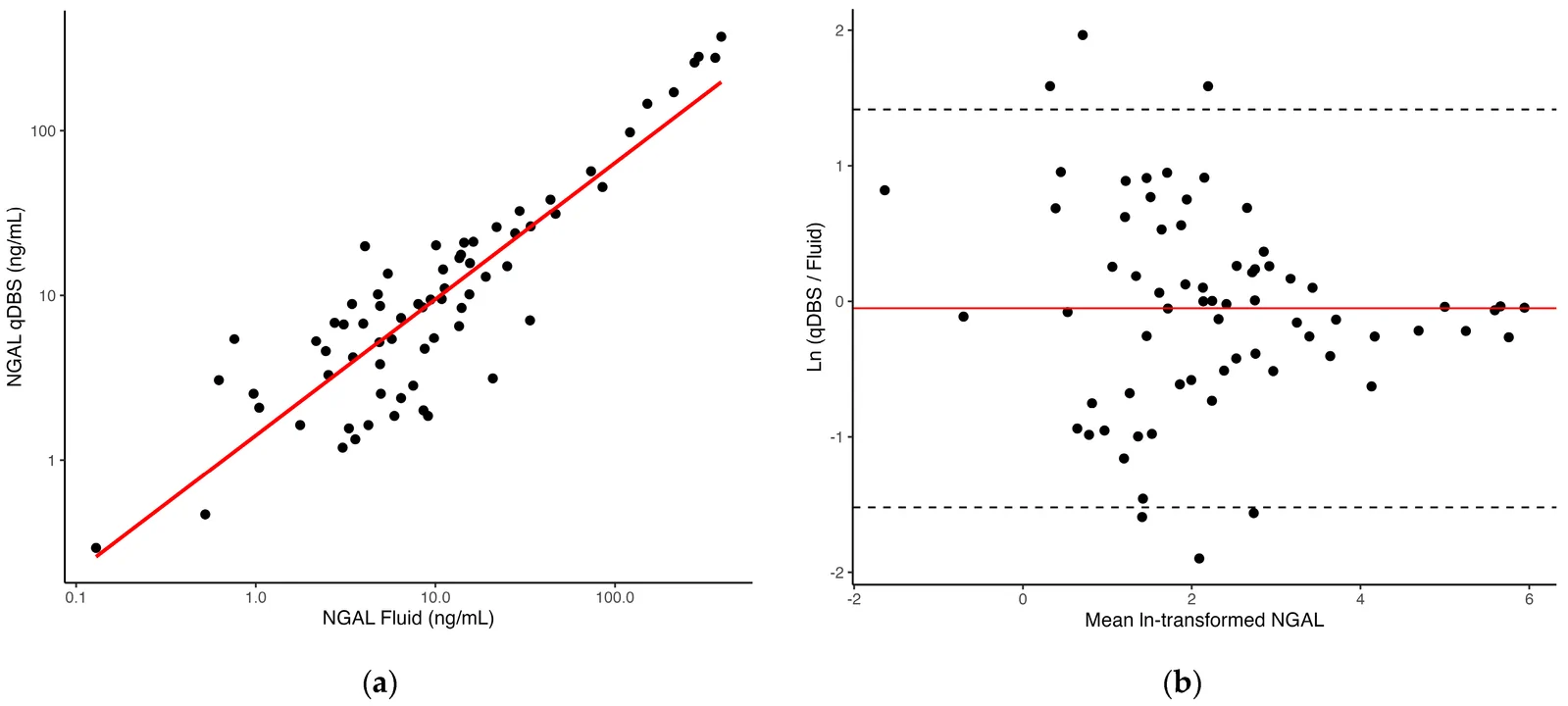
(**a**) Correlation between NGAL concentrations measured in liquid urine and in eluates from Capitainer qDBS discs. Each point represents one urine sample (*n* = 69). Linear regression showed a strong relationship between the two methods (R^2^ = 0.98), described by qDBS = 0.88 × liquid urine [ng/mL] − 0.089 [ng/mL]. Both axes are displayed on a logarithmic scale. The regression line was calculated using the original (non-transformed) NGAL concentrations. (**b**) Bland–Altman plot based on logarithmically transformed NGAL concentrations measured in liquid urine and qDBS eluates (*n* = 69). The solid line indicates the mean log difference and the dashed lines represent the 95% limits of agreement.

Linear regression between NGAL concentrations measured from qDBS eluates showed a strong correlation with liquid urine measurements between the two methods (R^2^ = 0.98), described by the equation qDBS = 0.88 × liquid urine [ng/mL] − 0.089 [ng/mL] (Figure 1a). Passing–Bablok regression showed no evidence of constant bias (intercept 0.91, 95% CI −0.03 to 1.76), but indicated a small proportional bias (slope 0.88, 95% CI 0.79–0.95). Bland–Altman analysis showed good agreement across the measurement range, with a mean ratio of 0.95 and 95% limits of agreement from 0.22 to 4.13 (Figure 1b).

**Figure 2:**
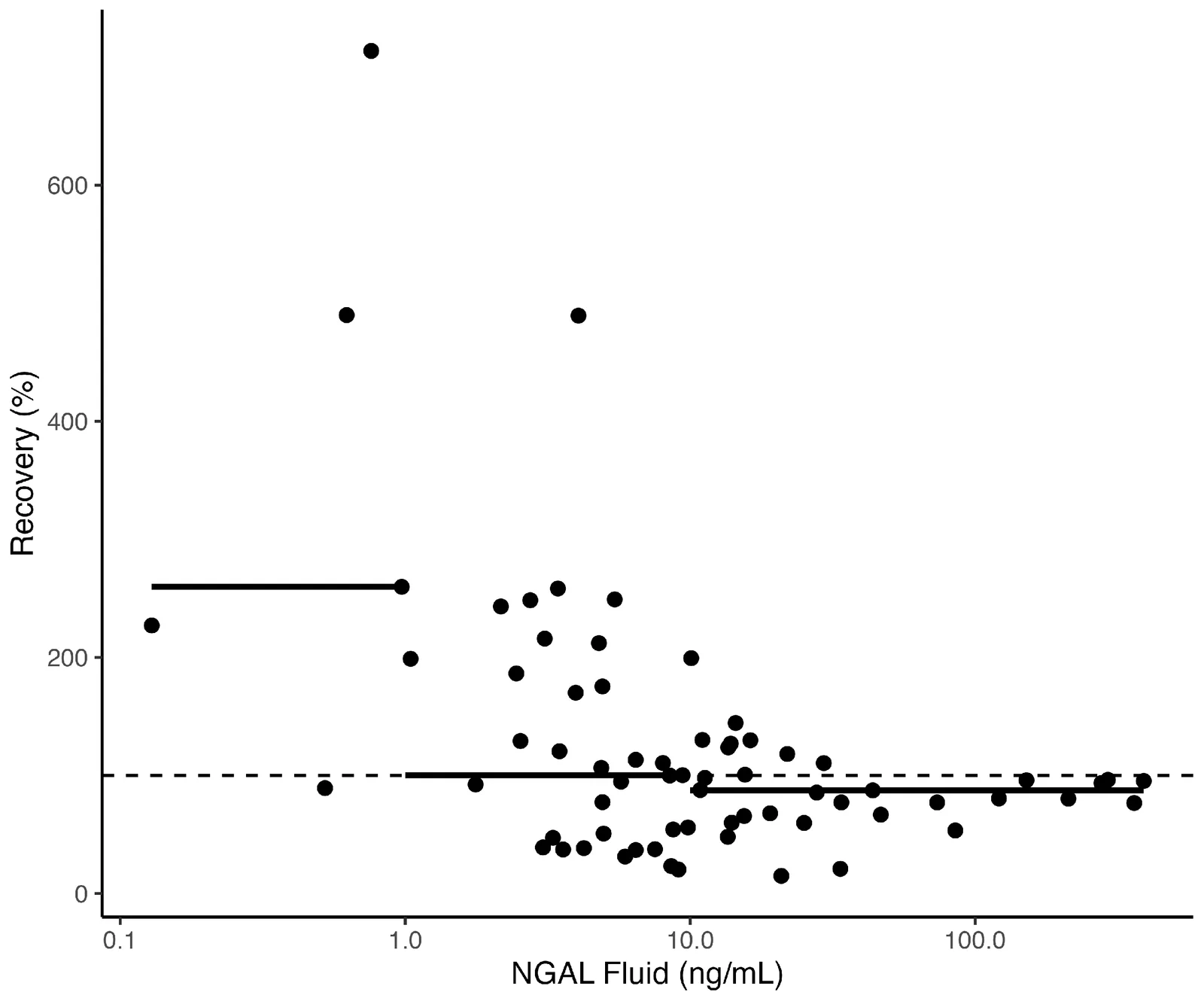
Recovery of NGAL measured from qDBS relative to liquid urine across the concentration range (*n* = 69). Each point represents one urine sample. The dashed horizontal line indicates 100% recovery. Solid horizontal lines indicate the median recovery within the concentration ranges <1 ng/mL (259.8%), 1–10 ng/mL (100.3%), and >10 ng/mL (87.4%).

The median recovery of NGAL from qDBS compared with liquid urine was 95.4% when calculated for samples with quantifiable concentrations in both methods. When all samples were included and values below the quantification limit were treated as zero for recovery calculations, the overall recovery was 90.9%. Recovery was concentration-dependent, with median recoveries of 259.8% for liquid urine NGAL concentrations < 1 ng/mL, 100.3% for concentrations of 1–10 ng/mL, and 87.4% for concentrations > 10 ng/mL. Recovery variability was greater at lower NGAL concentrations, whereas agreement was higher at higher concentrations (Figure 2).

## 4. Discussion

In this study, NGAL concentrations measured from dried urine samples collected using Capitainer qDBS discs showed good agreement with measurements obtained directly from liquid urine. A strong correlation and linear relationship were observed, and recovery was close to unity, with only a small negative bias.

The analytical performance observed in this study is consistent with expected characteristics of dried spot sampling. Slightly lower concentrations measured from Capitainer qDBS discs may reflect dilution during the elution step or partial adsorption of proteins to the filter material [4,9]. In line with this, a small number of samples yielded NGAL concentrations below the quantification limit in the qDBS method while remaining measurable in liquid urine. This difference was confined to very low NGAL concentrations and likely reflects reduced analytical sensitivity related to the smaller effective sample volume and the elution process.

In addition, uneven distribution of analytes within dried spots has been described and may affect quantitative accuracy, particularly in non-volumetric sampling approaches [9,10]. Although volumetric sampling reduces this variability, it may still contribute to lower measured concentrations or false negative results at low analyte levels.

In pediatric clinical practice, repeated urine sampling is often required in children with chronic kidney and urinary tract conditions. These patients frequently attend healthcare facilities for monitoring, placing a considerable logistical and psychosocial burden on families, particularly those living in rural or remote areas with limited access to specialised care [11]. Simplified sampling strategies that allow for home collection, using a fixed-volume urine sampling device that can be dried at room temperature and subsequently transported to the laboratory, may improve the feasibility and acceptability of repeated sampling, thereby reducing logistical barriers for caregivers while maintaining sample quality [12]. In the present study, the observed agreement between Capitainer qDBS discs and liquid urine measurements suggests that such approaches may be applicable to urinary protein biomarkers. In addition, dried sampling eliminates the need for cold-chain transport and frozen storage, which may simplify logistics and reduce associated costs compared with conventional liquid sample handling. Although these are well-recognized advantages of dried matrix sampling, the stability of NGAL in dried urine under ambient storage conditions was beyond the scope of the present study. This may support multicenter studies and facilitate sample handling in settings where conventional infrastructure is limited [13]. Enabling collection outside clinical settings and transport without cold-chain requirements, opens up possibilities for diagnostic testing and research logistics in resource-limited environments [14].

A strength of this study is the use of clinical urine samples across a range of NGAL concentrations, allowing evaluation of method performance under realistic conditions. However, the number of samples was relatively small and the NGAL concentrations covered a limited range, which may affect generalizability. In addition, only one biomarker and one analytical platform were evaluated. Pre-analytical handling was standardized, with rapid transport under cooled conditions before urine was applied to the qDBS device. However, the robustness of the dried sampling approach under varying pre-analytical conditions, such as delayed processing or ambient storage prior to analysis, was not evaluated in this study. Furthermore, urine is a heterogeneous biological matrix, and characteristics such as osmolality, protein content, specific gravity and pH may influence sample absorption and analyte recovery, and these factors were not evaluated in the present study. Finally, the use of clinical leftover samples may introduce heterogeneity in sample characteristics, although it also reflects real-world conditions.

## 5. Conclusions

In conclusion, NGAL can be quantified from dried urine samples collected using Capitainer qDBS discs, with good agreement compared with liquid urine measurements. These findings indicate that volumetric dried urine sampling can provide quantitatively comparable results for urinary protein biomarkers. Dried urine sampling may therefore represent a practical alternative for studies requiring simplified sample handling, decentralized collection, or large-scale logistics.

## Data Availability

The data presented in this study are available on request from the corresponding author. The data are not publicly available due to ethical restrictions related to the use of clinical patient data.

## Abbreviations

The following abbreviations are used in this manuscript:

NGAL	Neutrophil gelatinase-associated lipocalin
qDBS	Quantitative dried blood spot
ELISA	Enzyme-linked immunosorbent assay
PBS	Phosphate-buffered saline
IQR	Interquartile range
CI	Confidence interval
